# Ultrawidefield OCTA study for the impact of PPVP on the structure and blood flow of the retina and choroid

**DOI:** 10.3389/fmed.2026.1790917

**Published:** 2026-04-24

**Authors:** Xida Liang, Jing Zeng, Ruiming Yang, Wanni Chen, Zhimeng Zhang, Junming Wang

**Affiliations:** 1 Ophthalmic Center, The Second Affiliated Hospital, Guangzhou Medical University, Guangzhou, Guangdong, China; 2 Department of ophthalmology, Guangzhou First People’s Hospital, the Second Affiliated Hospital of South China University of Technoloty, Guangzhou, China

**Keywords:** blood flow, choroid, OCTA, PPVP, retina

## Abstract

**Objective:**

To investigate the association between the posterior precortical vitreous pocket (PPVP) and retinal morphological and blood flow parameters, with the aim of elucidating its potential pathophysiological significance.

**Methods:**

This prospective single-center study included 65 healthy subjects. PPVP was assessed using spectral-domain optical coherence tomography (OCT) and optical coherence tomography angiography (OCTA). The associations between PPVP presence and macular retinal thickness, volume, and vessel density were analyzed.

**Results:**

PPVP was detected in 72.3% (47/65) of subjects. Compared with the PPVP-negative group, the PPVP-positive group showed significantly greater retinal thickness in the superior inner macula (362.91 ± 16.84 vs. 344.06 ± 15.59 μm, *p* < 0.001) and nasal inner macula (361.96 ± 16.83 vs. 348.00 ± 15.45 μm, *p* = 0.003). The PPVP-positive group also had a larger volume in the superior inner macula (0.58 ± 0.05 vs. 0.54 ± 0.06, *p* = 0.001). In addition, vessel density was significantly higher in the PPVP-positive group in the superficial nasal retina (34.13 ± 7.88 vs. 23.83 ± 11.09, *p* = 0.001) and deep nasal retina (36.66 ± 6.58 vs. 25.44 ± 13.83, *p* = 0.004).

**Conclusion:**

The presence of PPVP was significantly associated with localized retinal thickening, increased retinal volume, and enhanced retinal blood flow. These findings suggest that PPVP may influence the vitreoretinal interface through biomechanical or microcirculatory mechanisms. This study systematically characterizes the morphological and functional features associated with PPVP and may contribute to a better understanding of the physiological vitreoretinal interface, with potential implications for future research into related retinal disorders.

## 1 Introduction

The vitreous is a transparent, gel-like substance that fills most of the posterior segment of the eye. Its primary functions are to maintain ocular shape, transmit light, and facilitate intraocular metabolic transport. The mechanical interface between the vitreous and the retina plays a crucial role in the pathogenesis of conditions such as macular holes and epiretinal membranes. Studies have shown that the vitreous is not a homogeneous structure. In 1814, Martegiani described cavity-like structures anterior to the optic disc and retina, which were termed the “bursa premacularis” (1). Later, Kishi renamed this structure the posterior precortical vitreous pocket (PPVP) (2).

Although PPVP is recognized as a normal anatomical structure, it has attracted considerable research interest because of its location at the vitreoretinal interface, a region critically involved in numerous pathological conditions. Accumulating evidence suggests that alterations in PPVP morphology may be associated with various macular diseases. For example, PPVP characteristics have been linked to the manifestations of proliferative diabetic retinopathy (3), and larger PPVP volumes have been observed in eyes with macular holes (4). Potential associations have also been reported with optic disc pit maculopathy (5) and idiopathic preretinal macular fibrosis (6). Therefore, although the physiological role of PPVP remains incompletely understood, its potential relationship with retinal parameters in both health and disease warrants further investigation. This study aimed to systematically explore the association between PPVP and quantitative retinal parameters in a healthy cohort.

In recent years, advances in optical coherence tomography (OCT) technology have shown that PPVP is commonly present in healthy individuals, leading to a clearer understanding of its morphological features. Existing literature has focused mainly on the morphological description and detection rate of PPVP. Multiple cross-sectional studies have reported that the detection rate of PPVP in adults can reach 60–80%, whereas it is relatively less common in children and adolescents (7–9). This observation suggests that PPVP formation may be related to age-dependent vitreous liquefaction. Technologically, high-resolution OCT and OCTA have become the gold standard for evaluating PPVP, enabling noninvasive visualization of its three-dimensional structural characteristics. However, important gaps remain in our understanding of the physiological function of PPVP and its interactions with retinal microstructure, particularly with respect to regional associations.

In our clinical observations, PPVP was detectable by high-resolution OCT in most healthy individuals included in this study. A small subset lacked a discernible PPVP structure. We therefore sought to determine whether the anatomical presence of PPVP is associated with localized retinal structural remodeling and whether its presence influences regional retinal blood flow distribution. Specifically, we investigated whether the presence or absence of PPVP is associated with differences in the vitreoretinal interface environment, as reflected by variations in retinal and choroidal structure and blood flow. Accordingly, we systematically evaluated the associations between PPVP and retinal and choroidal parameters using high-definition OCT and OCTA.

## 2 Method

This prospective single-center study was approved by the Institutional Review Board of the Second Affiliated Hospital of Guangzhou Medical University. The study adhered to the principles of the Declaration of Helsinki and relevant regulations of the National Health Commission. A total of 65 right eyes from 65 healthy participants were recruited from individuals presenting for routine ocular examinations at our clinic.

The inclusion criterion was the availability of sufficiently high-quality OCTA scans (scan quality score ≥ 6) covering a 24 × 20 mm area. Exclusion criteria included poor image quality (scan quality score < 6), media opacity, and scan artifacts caused by blinking or eye movements that interfered with PPVP assessment.

The posterior precortical vitreous pocket (PPVP) is a specialized structure within the posterior vitreous and typically appears as a boat-shaped or oval liquefied space anterior to the macular region. Demographic and clinical data, including age, sex, lens status, and the presence of high myopia, were collected through chart review. All participants underwent comprehensive ophthalmic examinations, including Snellen visual acuity assessment, anterior segment biomicroscopy, dilated fundus examination, spectral-domain OCT, and OCTA (Tupai Corporation).

Participants underwent 24 × 20 mm volumetric scans using the spectral-domain Solix device, which generated cross-sectional B-scans, en face OCT images, and 20-mm-wide × 6.5-mm-deep OCT B-scans. Ultra-widefield OCTA was deliberately selected to optimize the accuracy and reliability of the initial and critical step of PPVP assessment. OCTA was also used to obtain retinal thickness and vessel density measurements in the macular and optic disc regions. All images were evaluated for the presence, grade, and location of PPVP and posterior vitreous detachment (PVD).

All quantitative OCTA-derived parameters, including macular vessel density, retinal thickness, and retinal volume, were tested for normality using the Shapiro–Wilk test. Because the data followed a normal distribution, intergroup comparisons between PPVP-positive and PPVP-negative subjects were performed using independent-samples *t* tests. All analyses were conducted using SPSS software, and a two-sided *p* value < 0.05 was considered statistically significant before correction. To account for multiple comparisons across the nine macular subfields, a Bonferroni correction was applied, and an adjusted *p* value < 0.0056 (0.05/9) was considered statistically significant.

## 3 Results

**Figure 1:**
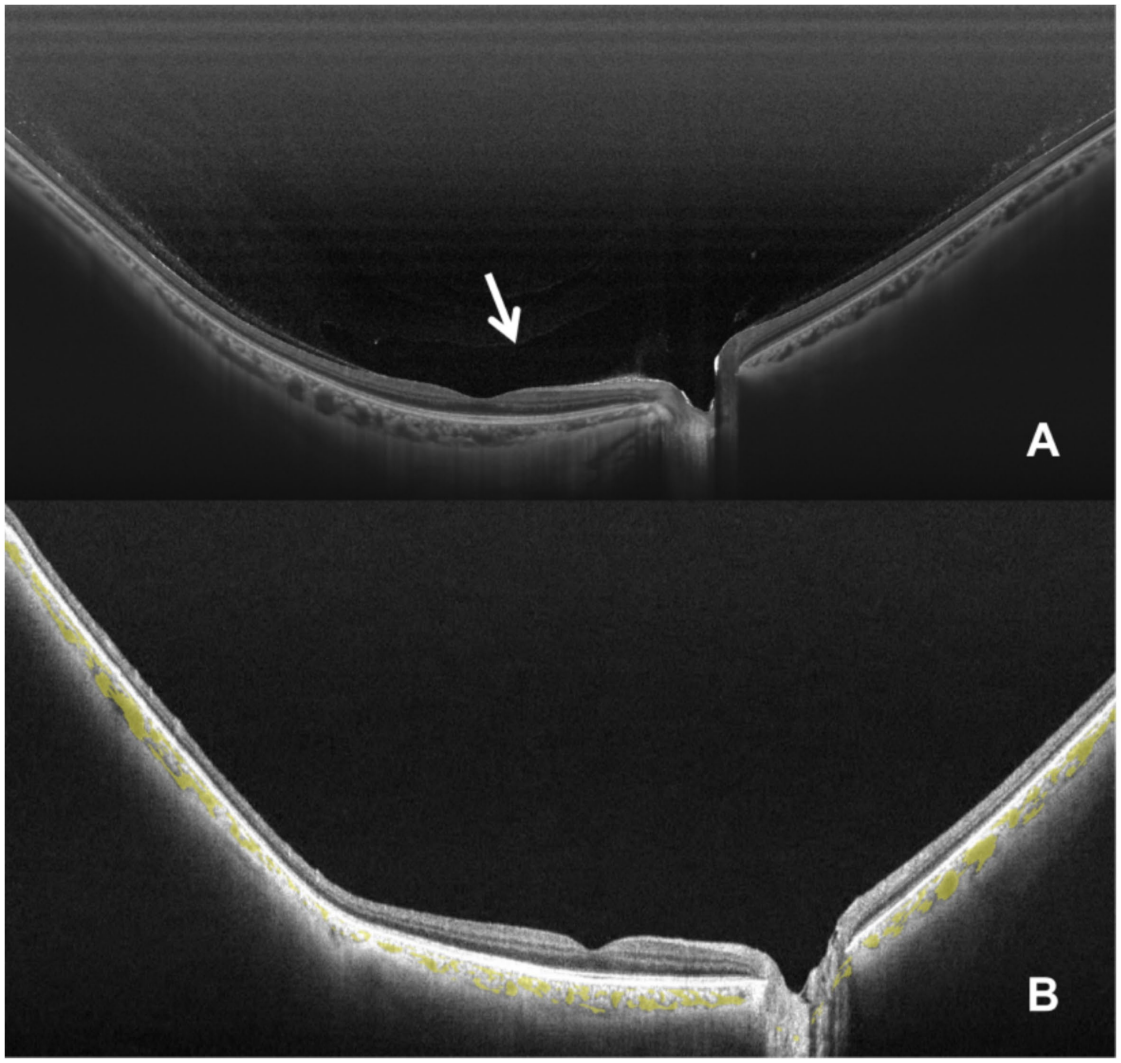
Swept-source deep range imaging optical coherence tomography of the cortical vitreous. Measurement of the posterior precortical vitreous pocket (PPVP). **(A)** PPVP-positive cortical vitreous. **(B)** PPVP-negative cortical vitreous.

This study included 65 eyes from 65 participants. Among them, 47 eyes were classified as PPVP-positive and 18 as PPVP-negative (Figure 1). Baseline characteristics did not differ significantly between the two groups (Table 1).

**Table 1:** Clinical characteristics of the populations.

Characteristics	PPVP group	nPPVP group	T	*p*
No. of eyes	47	18		
Female sex, no. (%)	25 (53.1)	10 (55.6)		
Age, mean±SD	33.28 ± 17.33	32.56 ± 14.23	−0.157	0.876

PPVP, Posterior precortical vitreous pocket; SD, Standard deviation.

In terms of thickness parameters, overall foveal retinal and choroidal thicknesses did not differ significantly between groups. However, the PPVP-positive group had greater retinal thickness in the superior inner macula (362.91 ± 16.84 μm) than the PPVP-negative group (344.06 ± 15.59 μm, *p* < 0.001). Retinal thickness in the nasal inner macula was also greater in the PPVP-positive group (361.96 ± 16.83 μm) than in the PPVP-negative group (348.00 ± 15.45 μm, *p* = 0.003). No statistically significant differences were observed in the inferior inner macula, temporal inner macula, or the temporal, superior, nasal, and inferior outer macular regions (Table 2).

**Table 2:** Retinal and choroid thickness in PPVP and nPPVP group.

Characteristics	PPVP group	nPPVP group	T	*p*	Bonferroni correction (*α*’ = 0.00556)
Macula foveal thickness	282.72 ± 18.38	275.83 ± 13.23	−1.45	0.152	
Temporal thickness of inner circle macula	345.68 ± 14.66	337.33 ± 17.43	−1.948	0.056	
Superior thickness of inner circle macula	362.91 ± 16.84	344.06 ± 15.59	−4.12	<0.001	*
Nasal thickness of inner circle macula	361.96 ± 16.83	348 ± 15.45	−3.058	0.003	*
Inferior thickness of inner circle macula	354.96 ± 15.98	349.06 ± 18.89	−1.266	0.21	
Temporal thickness of outer circle macula	300.7 ± 14.92	296.56 ± 14.54	−1.01	0.317	
Superior thickness of outer circle macula	322.47 ± 15.23	317.11 ± 21.7	−0.961	0.346	
Nasal thickness of outer circle macula	334 ± 16.86	334.72 ± 20.73	0.145	0.885	
Inferior thickness of outer circle macula	303.19 ± 12.6	301.22 ± 15.09	−0.533	0.596	

Regarding volume parameters, overall foveal retinal and choroidal volumes did not differ significantly between the two groups. However, the PPVP-positive group had a larger volume in the superior inner macula (0.58 ± 0.05) than the PPVP-negative group (0.54 ± 0.06, *p* = 0.001). No significant differences were observed in the temporal, nasal, or inferior inner macula or in any of the outer macular subfields (Table 3).

**Table 3:** Retinal and choroid volume in PPVP and nPPVP group.

Characteristics	PPVP group	nPPVP group	T	*p*	Bonferroni correction (*α*’ = 0.00556)
Macula foveal volume	0.24 ± 0.04	0.24 ± 0.05	0.19	0.85	
Temporal volume of inner circle macula	0.54 ± 0.02	0.53 ± 0.03	−1.541	0.128	
Superior volume of inner circle macula	0.58 ± 0.05	0.54 ± 0.06	−3.367	0.001	*
Nasal volume of inner circle macula	0.58 ± 0.05	0.55 ± 0.06	−1.942	0.057	
Inferior volume of inner circle macula	0.56 ± 0.03	0.55 ± 0.03	−1.742	0.086	
Temporal volume of outer circle macula	1.35 ± 0.43	1.34 ± 0.42	−0.134	0.894	
Superior volume of outer circle macula	1.47 ± 0.43	1.45 ± 0.42	−0.224	0.823	
Nasal volume of outer circle macula	1.54 ± 0.44	1.54 ± 0.44	0.046	0.964	
Inferior volume of outer circle macula	1.4 ± 0.39	1.4 ± 0.38	−0.003	0.998	

**Figure 2:**
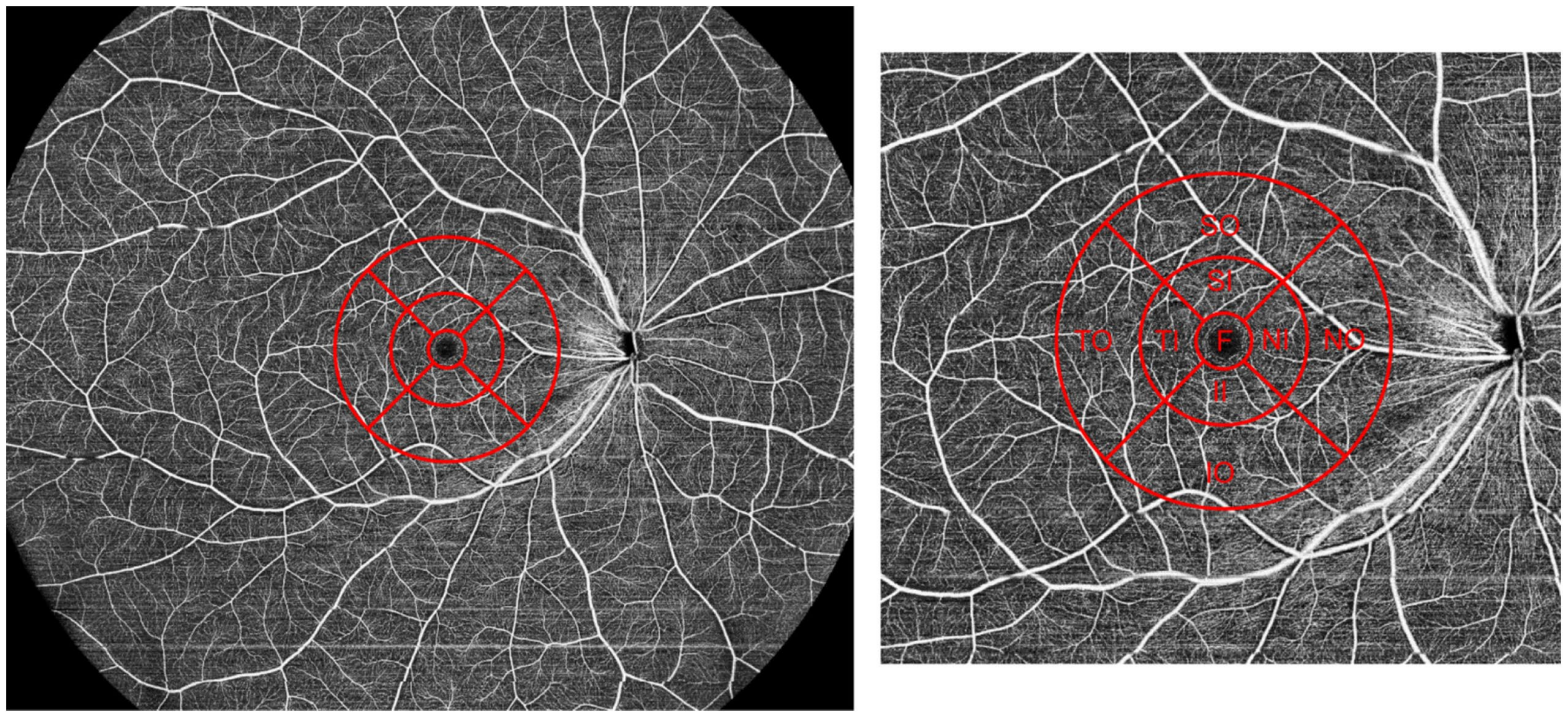
Schematic diagram of the 24 × 20 mm OCTA scan and subfield division. F: Macula foveal; SI: superior part of inner circle macula; NI: nasal part of inner circle macula; II: inferior part of inner circle macula; TI: temporal part of inner circle macula; SO: superior part of outer circle macula; NO: nasal part of outer circle macula; IO: inferior part of outer circle macula; TO: temporal part of outer circle macula.

For vessel density parameters, overall macular retinal and choroidal blood flow did not differ significantly between groups. However, the PPVP-positive group exhibited higher superficial retinal vessel density in the nasal inner macula (34.13 ± 7.88) than the PPVP-negative group (23.83 ± 11.09, *p* = 0.001). Similarly, deep retinal vessel density in the nasal inner macula was higher in the PPVP-positive group (36.66 ± 6.58) than in the PPVP-negative group (25.44 ± 13.83, *p* = 0.004). These differences remained significant after Bonferroni correction (Figure 2).

In addition, several other subfields showed trends toward intergroup differences, although these did not remain significant after Bonferroni correction. These included deep vessel density in the outer nasal macula (39.72 ± 6.47 vs. 31.00 ± 14.18, *p* = 0.021), and deep vessel density in the outer inferior macula (40.87 ± 6.63 vs. 36.44 ± 7.63, *p* = 0.024). No statistically significant differences were observed in superficial or deep vessel density in the temporal quadrants of the inner or outer macula. No significant differences were found in choroidal vessel density in any macular quadrant (Table 4).

**Table 4:** Retinal and choroid blood flow density in PPVP and nPPVP group.

Characteristics	PPVP group	nPPVP group	T	*p*	Bonferroni correction (*α*’ = 0.00556)
Macular superficial retinal blood flow density	21.02 ± 9.01	18.17 ± 12.8	−0.868	0.394	
Temporal superficial retinal blood flow density of inner circle macular	29 ± 9.84	24.17 ± 11.75	−1.679	0.098	
Superior superficial retinal blood flow density of inner circle macular	32.68 ± 8.96	28.83 ± 10.76	−1.465	0.148	
Nasal superficial retinal blood flow density of inner circle macular	34.13 ± 7.88	23.83 ± 11.09	−3.604	0.001*	*
Inferior superficial retinal blood flow density of inner circle macular	34.79 ± 6.46	30.39 ± 12.58	−1.414	0.172	
Temporal superficial retinal blood flow density of outer circle macular	38.64 ± 5.75	34.5 ± 8.87	−1.836	0.079	
Superior superficial retinal blood flow density of outer circle macular	42.04 ± 4.35	41.11 ± 5.17	−0.733	0.467	
Nasal superficial retinal blood flow density of outer circle macular	40.77 ± 5.26	38.22 ± 4.83	−1.782	0.08	
Inferior superficial retinal blood flow density of outer circle macular	41.6 ± 5.14	39.28 ± 6.34	−1.524	0.133	
Macular deep retinal blood flow density	15.3 ± 10.66	13.33 ± 10.47	−0.668	0.507	
Temporal deep retinal blood flow density of inner circle macular	31.36 ± 10.88	25.44 ± 12.87	−1.865	0.067	
Superior deep retinal blood flow density of inner circle macular	35.06 ± 9.69	28.72 ± 13.41	−1.831	0.079	
Nasal deep retinal blood flow density of inner circle macular	36.66 ± 6.58	25.44 ± 13.83	−3.301	0.004*	*
Inferior deep retinal blood flow density of inner circle macular	37.15 ± 8.27	32 ± 14.92	−1.385	0.18	
Temporal deep retinal blood flow density of outer circle macular	38.7 ± 8.4	34.89 ± 9.92	−1.557	0.124	
Superior deep retinal blood flow density of outer circle macular	39.98 ± 5.84	37.94 ± 9.61	−0.841	0.41	
Nasal deep retinal blood flow density of outer circle macular	39.72 ± 6.47	31 ± 14.18	−2.513	0.021*	
Inferior deep retinal blood flow density of outer circle macular	40.87 ± 6.63	36.44 ± 7.63	−2.31	0.024*	
Macular superficial choroid blood flow density	39.94 ± 8.95	41.72 ± 4.82	1.032	0.307	
Temporal superficial choroid blood flow density of inner circle macular	40.23 ± 7.1	39.44 ± 4.5	−0.438	0.663	
Superior superficial choroid blood flow density of inner circle macular	40.72 ± 7.46	40.33 ± 4.34	−0.208	0.836	
Nasal superficial choroid blood flow density of inner circle macular	40.64 ± 7.33	40.72 ± 5.53	0.044	0.965	
Inferior superficial choroid blood flow density of inner circle macular	41.7 ± 5.63	41 ± 4.19	−0.48	0.633	
Temporal superficial choroid blood flow density of outer circle macular	43.26 ± 6.22	42.39 ± 4.29	−0.542	0.59	
Superior superficial choroid blood flow density of outer circle macular	43.21 ± 4.88	42.78 ± 4.56	−0.327	0.745	
Nasal superficial choroid blood flow density of outer circle macular	44.02 ± 4.97	42.44 ± 5.01	−1.143	0.257	
Inferior superficial choroid blood flow density of outer circle macular	42.4 ± 6.02	43.67 ± 3.94	0.988	0.328	
Macular deep choroid blood flow density	48.72 ± 7.69	48.56 ± 10.35	−0.071	0.943	
Temporal deep choroid blood flow density of inner circle macular	49.21 ± 7.92	47 ± 9.53	−0.952	0.345	
Superior deep choroid blood flow density of inner circle macular	48.79 ± 8.54	49.33 ± 8.24	0.233	0.817	
Nasal deep choroid blood flow density of inner circle macular	50.21 ± 8.37	46.44 ± 10.86	−1.492	0.141	
Inferior deep choroid blood flow density of inner circle macular	49.04 ± 7.27	47.22 ± 9.53	−0.827	0.411	
Temporal deep choroid blood flow density of outer circle macular	48.94 ± 8.03	48.11 ± 6.4	−0.391	0.697	
Superior deep choroid blood flow density of outer circle macular	48.62 ± 7.12	48.89 ± 6.61	0.14	0.889	
Nasal deep choroid blood flow density of outer circle macular	50.3 ± 8.32	49.28 ± 9.53	−0.425	0.672	
Inferior deep choroid blood flow density of outer circle macular	49.49 ± 7.59	49.28 ± 7.78	−0.1	0.921	

## 4 Discussion

The posterior precortical vitreous pocket (PPVP) is an important anatomical feature of the vitreous and has recently attracted increasing attention because of its potential role in vitreoretinal interface disorders. Located between the posterior vitreous cortex and the retina, morphological and functional changes in PPVP have been associated with several ocular pathologies, including high myopia, posterior vitreous detachment (PVD), and retinal detachment (10–12). With advances in optical coherence tomography (OCT), particularly swept-source OCT (SS-OCT), the morphology of PPVP and its dynamic relationship with surrounding structures can now be visualized more clearly (11, 13). In the present study, subjects were categorized according to the presence or absence of a detectable PPVP, and the associations between PPVP status and anatomical and hemodynamic changes in the retina and choroid were analyzed.

On SS-OCT, PPVP typically appears as a boat-shaped, oval, or hook-shaped liquefied space anterior to the macular region and may communicate with Cloquet’s canal through connecting channels (8, 10). The posterior wall of the PPVP consists of a thin layer of vitreous cortex and is thinnest over the macula (11). Furthermore, PPVP morphology has been reported to vary with body position; for example, when changing from a sitting to a supine position, the anterior border of the PPVP moves anteriorly and the superior portion expands (14). Age is another important determinant of PPVP morphology. Previous studies have shown that PPVP width is positively correlated with age, and individuals older than 50 years tend to have significantly wider PPVPs than younger individuals (1). In children, PPVP becomes detectable from approximately 3 years of age and gradually enlarges thereafter, with both depth and width significantly correlated with age (*p* < 0.001) (15). In addition, the channels connecting PPVP and Cloquet’s canal usually begin to form after 5 years of age and increase in number over time (9). The onset of partial or complete PVD also occurs significantly earlier in highly myopic eyes than in non-myopic eyes (*p* < 0.0001) (16). Moreover, abnormal PPVP morphology may be associated with high-myopia-related macular disorders such as macular holes (16).

In the present study, a subset of participants lacked a visible PPVP and was therefore classified into the PPVP-negative group. Compared with this group, the PPVP-positive group showed significantly greater superficial and deep retinal vessel density in the nasal macula. Given that PPVP is primarily located anterior to the macular retina and optic disc, this regional association may be anatomically meaningful. One possible explanation is that the presence of a liquefied PPVP alters local biomechanical conditions at the vitreoretinal interface. It is conceivable that such a pocket may act as a fluid buffer and thereby modulate local mechanical stress transmission from the vitreous body. However, this interpretation remains speculative and does not fully explain the observed sector-specific associations. The mechanistic link between PPVP morphology and retinal hemodynamics therefore requires further investigation.

Previous studies have shown that PPVP morphology changes with body position; for example, when the body shifts from sitting to supine, the anterior border moves forward and the superior portion expands (14). These findings suggest that the fluid-filled PPVP is capable of dynamic deformation in response to pressure direction, which may be relevant to the maintenance of normal retinal hemodynamics. In our study, the significant difference in nasal blood flow between groups was consistent with the anatomical location of PPVP, raising the possibility that PPVP may have a cushioning or buffering effect associated with local retinal perfusion.

The absence of a visible PPVP may also be related to a different stage or pattern of posterior vitreous detachment. Previous studies have shown that PVD progression can be divided into several stages, ranging from paramacular detachment of the posterior wall of the PPVP (stage 1) to complete PVD (stage 4) (12). The anatomical features of PPVP may facilitate the development of PVD, particularly in highly myopic eyes, in which an enlarged PPVP may accelerate the early onset of PVD (16). In addition, the channels connecting PPVP and Cloquet’s canal may represent sites of early vitreous liquefaction, thereby contributing to PVD progression (11).

The morphology of PPVP also has potential implications for vitreoretinal surgery. For example, in minimally invasive vitrectomy, induction of PVD through the PPVP may reduce mechanical retinal injury (17). In addition, abnormal traction related to PPVP may contribute to optic disc pit maculopathy, and release of this traction during surgery may improve retinal reattachment and visual outcomes (5). Using ultra-widefield OCT and three-dimensional reconstruction, previous investigators have described complex spatial relationships among PPVP, Cloquet’s canal, previtreal fissures (PVFs), and vitreous cisterns (18, 19). These structures are often distributed around retinal vessels and overlap with areas of strong vitreoretinal adhesion (19–21). Such spatial organization may help explain the dynamic role of PPVP in vitreous liquefaction and PVD.

To our knowledge, this is the first study to systematically evaluate the association between PPVP and macular retinal structural and hemodynamic parameters, thereby addressing a gap in the quantitative analysis of regional variation in this field. Our finding that eyes without a detectable PPVP exhibited altered macular vascular parameters suggests that the vitreous may play a previously underappreciated role in retinal homeostasis. The vitreous is known to be influenced by systemic conditions; for example, acute hypoxia can induce structural and functional ocular changes, including alterations in retinal blood flow (22). Likewise, age-related vitreous liquefaction and posterior vitreous detachment have been epidemiologically linked to vascular retinopathies (23). These observations suggest that the vitreous may act as an intermediary or cofactor through which systemic or age-related factors influence the retinal microvasculature. As a premacular liquefied space, PPVP may modulate this interaction. Its absence may reflect a distinct biomechanical or biochemical environment at the vitreoretinal interface, potentially contributing to the vascular differences observed in our study. This hypothesis offers a plausible interpretive framework and highlights an important direction for future longitudinal research.

Previous studies have focused primarily on the morphology of PPVP and its relationship with PVD, with limited attention to its association with localized retinal thickness, volume, and microcirculation. Using high-resolution OCTA, we found for the first time in a healthy population that the PPVP-positive group had significantly greater retinal thickness, volume, and vessel density in the superior and nasal inner macular regions than the PPVP-negative group. These findings suggest that PPVP may not be merely a passive anatomical structure, but rather may be associated with measurable differences in the local retinal microenvironment. This observation provides a new perspective for understanding early changes at the vitreoretinal interface.

This exploratory study further suggests that the presence of PPVP, a common anatomical feature, is associated with distinct macular imaging characteristics. From a clinical research perspective, this underscores the importance of accounting for normal anatomical variation when interpreting macular imaging data. Although these associations do not indicate pathology, they provide a rationale for including PPVP status as a potential anatomical covariate in future studies of individual variation in vitreoretinal interface anatomy and its possible long-term implications.

However, the increased retinal thickness and vessel density observed in areas corresponding to PPVP should be interpreted with caution. While these features may eventually prove relevant to disorders such as epiretinal membranes, macular holes, or retinal detachment, the present data do not support their use as predictive clinical indicators. Similarly, although OCTA is noninvasive and reproducible and may be useful for large-scale screening and longitudinal follow-up, the routine incorporation of PPVP assessment into standard ophthalmic examinations cannot be recommended on the basis of this exploratory study alone.

This study has several limitations. First, the sample size was relatively small, and the subgroup sizes were uneven, which may limit the statistical power and generalizability of the findings. Larger prospective studies are needed to validate these preliminary observations. Second, because of the cross-sectional design, causality cannot be inferred. Whether the presence of PPVP contributes to retinal thickening and increased blood flow, or whether these retinal characteristics are associated with PPVP detectability, remains to be determined in longitudinal studies.

In conclusion, this study systematically demonstrates a significant association between PPVP and localized retinal thickness, retinal volume, and vessel density, providing new evidence regarding physiological interactions at the vitreoretinal interface. The finding that PPVP is mainly associated with the nasal and superior macular regions suggests that the posterior vitreous cortex may influence the retinal microenvironment through region-specific mechanisms. In clinical practice, for the relatively bigger proportion of individuals with PPVP, it may be worthwhile to pay attention to their retinal structural and hemodynamic characteristics and consider whether these traits might exert certain influences on retinal or glaucomatous diseases. Future studies should further investigate the molecular and biomechanical mechanisms underlying PPVP and explore its potential relevance to the pathogenesis and management of vitreoretinal disorders.

## Data Availability

The original contributions presented in the study are included in the article/supplementary material, further inquiries can be directed to the corresponding author.
